# Supplementary material for: P2X Purinergic Receptors Are Multisensory Detectors for Micro-Environmental Stimuli That Control Migration of Tumoral Endothelium
Source: Cancers (Basel). 2022 May 31;14(11):2743. doi: 10.3390/cancers14112743 (PMC9179260; doi:10.3390/cancers14112743)
Supplement: Supplementary file 1 [file cancers-14-02743-s001.zip › cancers-1753459-supplementary.pdf]

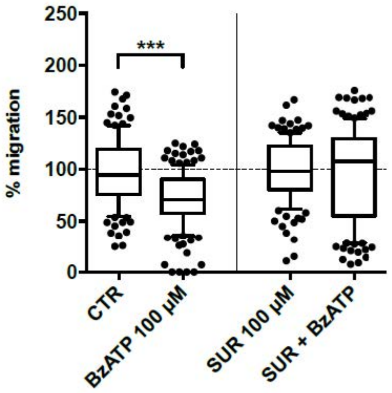

Figure S1. Percentage of BTEC migration in wound healing experiments at 12 hours after the treatment with 100 μM BzATP, 100 μM Suramin (SUR) or BzATP and Suramin together. Data are expressed as box and whiskers showing the median and 10-90 percentiles of 3 independent experiments for each condition ($n \geq 90$), each normalized to the corresponding control (CTR or SUR). Mann-Whitney test comparing BzATP treatment to the corresponding control. *** p-value < 0.0005.
